# Immunometabolic Dysfunction of Natural Killer Cells Mediated by the Hypoxia-CD73 Axis in Solid Tumors

**DOI:** 10.3389/fmolb.2019.00060

**Published:** 2019-07-24

**Authors:** Andrea M. Chambers, Sandro Matosevic

**Affiliations:** ^1^Department of Industrial and Physical Pharmacy, Purdue University, West Lafayette, IN, United States; ^2^Center for Cancer Research, Purdue University, West Lafayette, IN, United States

**Keywords:** NK cells, adenosine, CD73, solid tumors, immunometabolism, hypoxia

## Abstract

NK cell infiltration into solid tumors is often low and is largely represented by the poorly-cytotoxic CD56^bright^ subset. Numerous studies have demonstrated that CD73, overexpressed under conditions of hypoxia, is involved in a variety of physiological processes, while its overexpression has been correlated with tumor invasiveness, metastasis and poorer patient survival in many cancers. Hypoxia itself favors aggressive glycolytic fueling of cancer cells, in turn driving reprogramming of NK cell metabolism. In addition, the hypoxia-driven activity of CD73 immunometabolically impairs NK cells in tumors, due to its catalytic role in the generation of the highly immunosuppressive metabolite adenosine. Adenosinergic signaling was shown to alter NK cell metabolic programs, leading to tumor-promoting environments characterized by NK cell dysfunction. Despite the demonstrated role of NK cell responses in the context of CD73 targeting, the engagement of NK cells in the setting of hypoxia/CD73 signaling has not been extensively studied or exploited. Here, we discuss available evidence on the role of hypoxic signaling on CD73-mediated activity, and how this relates to the immunometabolic responses of NK cells, with a particular focus on the therapeutic targeting of these pathways.

## Introduction

Solid malignancies are commonly characterized by severe tumor hypoxia, the incidence of which occurs as a direct consequence of elevated cancer cell proliferation, altered metabolism and impaired oxygen and nutrient transport due to abnormal tumor vasculature (Muz et al., 2015). Related to the presence of hypoxia is the overexpression of ecto-5'-nucleotidase (CD73). CD73 is expressed on various cell types, including tumor stromal cells, endothelial cells, tumor-associated regulatory T cells, dendritic cells, as well as many cancers (Antonioli et al., 2016).

**Figure 1:**
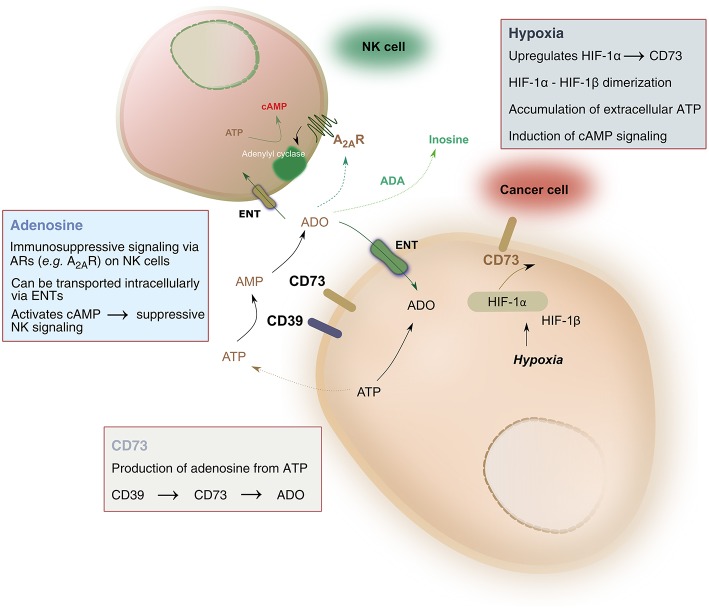
Hypoxia-CD73 signaling on NK cells. Hypoxia induces the expression of CD73 via HIF-1, itself consisting of HIF1-α and HIF-1β. HIF-1, in turn, regulates immune responses to hypoxia by controlling glycolytic metabolism and NK cells' adaptation to low oxygen. CD73 dephosphorylates AMP, produced by the conversion of ATP to AMP catalyzed by CD39, to generate adenosine. Extracellular adenosine can be transported intracellularly via equilibrative nucleoside transporters (ENT) or it can signal via adenosine receptors on the surface on NK cells, most notably the A_2A_ receptor (A_2A_R). A_2A_R signaling via adenosine was shown to result in dysfunction of NK cell metabolic and effector functions. Extracellular adenosine can also be converted to inosine via the catalytic activity of adenosine deaminase (ADA).

Pathophysiologic conditions of hypoxia, such as those found in solid tumors, drive significant metabolic changes in adenine nucleotides, such as adenosine triphosphate (ATP). Under hypoxic conditions, cancer cells release such adenine nucleotides in large amounts. Phosphohydrolysis of ATP is catalyzed by nucleoside triphosphate dephosphorylase (CD39), which converts ATP to adenosine monophosphate (AMP), followed by the activity of ecto-5′-nucleotidase (CD73) which hydrolyzes AMP to adenosine, ultimately liberating it into the extracellular space. As a result, adenosine accumulation in hypoxic tissues occurs largely due to the activity of CD39 and CD73 (Linden, 2001). The balance between ATP and adenosine concentration mediates immune homeostasis. Extracellular adenosine signals on immune cells, including natural killer (NK) cells, by binding to one of four adenosine receptors—most notably the A_2A_ receptor (A_2A_R)—and suppressing NK cell immune functions (Wang and Matosevic, 2018). As a result, the CD39-CD73-adenosine axis has been recognized as a strong immune regulator, due to the potent immunosuppressive functions of adenosine (Figure 1).

Hypoxia is a strong inducer of CD73 expression through the activity of hypoxia-inducible factor-1 (HIF-1). HIF-1 is a transcription factor which regulates immune responses to reduced O_2_ conditions (Semenza, 2012a). HIF-1 controls both O_2_ delivery and utilization through the regulation of oxidative and glycolytic metabolism, ultimately driving adaptation to oxygen-depleted environments (Shohet and Garcia, 2007; Halligan et al., 2016). Though important, HIF is not the exclusive regulator of metabolic responses on NK cells, as recent evidence has shown that priming of NK cells with the cytokines interleukin (IL)-2 and IL-12 requires cMyc but not HIF to drive metabolic responses (Loftus et al., 2018). Nonetheless, the roles of HIF-1 are many and varied, but its role in the control of cellular metabolism and the subsequent production of adenosine is what has received particular attention in cancer immunotherapy.

NK cells, a subset of innate lymphocytes, are sensitive to conditions of hypoxia (Ang et al., 2010; Velásquez et al., 2016). In the tumor microenvironment (TME), NK cells undergo significant metabolic reprogramming events which alter their phenotypic and functional responses (Chambers et al., 2018a; Dao and Matosevic, 2019). Their survival and cytotoxic function in hypoxic TMEs has been linked to the presence and activity of HIF-1α (Krzywinska et al., 2017). This is directly compounded by the accumulation of extracellular adenosine, which was shown to significantly alter NK activating receptors and metabolism by regulating glycolysis and oxidative phosphorylation (Raskovalova et al., 2005; Chambers et al., 2018b). In response to the known and growing potential of the adenosinergic cascade to cancer immunotherapy, multiple players within the adenosinergic signaling axis have been investigated as potential anti-cancer targets, including CD39 (Bonnefoy et al., 2015), A_2A_R (Young et al., 2016) and CD73 (Ghalamfarsa et al., 2019). Recently, we described the role of CD73 as a potential immune checkpoint target in the context of NK cell immunotherapy (Wang et al., 2018). Here, we discuss the mechanistic evidence for the immunometabolic regulation of NK cell function induced by hypoxia and CD73.

## Expression of CD73 in Solid Tumors

CD73 is predominantly a homodimer linked to the plasma membrane of a variety of cell types including cancer cells through a glycosylphosphatidylinositol (GPI) lipid linker, where it is largely involved in the metabolism of ATP. Though hypoxia is a formidable inducer of CD73 in cancer, CD73 expression on various cell types can be induced by a number of factors, including interferon-β-1a (Bellingan et al., 2014), the zinc finger transcription factor growth factor independence 1 (GFI-1) (Chalmin et al., 2012) and cyclic adenosine monophosphate (cAMP) response element binding protein (CREB) (Bao et al., 2016), as well as epigenetically (Wang et al., 2012), due to estrogen receptor expression (Spychala et al., 2004) or via Wnt signaling (Spychala and Kitajewski, 2004; Zhan et al., 2017).

It has largely been accepted that many cancers are characterized by overexpression of CD73. Wang and colleagues (Wang et al., 2017) determined that expression of CD73 was associated with poor overall survival and worse disease-free survival in many solid tumors, based on analysis of 1039 studies. More recently, Jiang et al. (2018) reported the comprehensive evaluation of CD73 expression on various cancers on the basis of a broad analysis of 12,533 patients. They found that CD73 overexpression was correlated with poor prognosis in cancers including bladder, brain, invasive lobular breast, esophageal, gastric, pancreatic, rectal mucinous, renal cell, lung large cell, oral squamous cell carcinoma, melanoma, and lung adenocarcinoma. This was corroborated by other studies in prostate (Leclerc et al., 2016), ovarian (Turcotte et al., 2015), colorectal (Wu et al., 2012), melanoma (Reinhardt et al., 2017), and triple negative breast (Buisseret et al., 2018) cancer, as well as oral squamous cell carcinoma (Ren et al., 2016). Elevated CD73 expression was also associated with lymph node metastases.

However, opposing views on the role of CD73 in cancer have been proposed. For instance, in the same analysis Jiang and colleagues reported no significant increase in CD73 expression in a number of cancers, namely cervical, liver, colorectal, prostate invasive ductal breast, small cell lung cancer and lung squamous cell carcinoma. Other studies have also reported that CD73 expression was correlated with a positive prognosis, for example in breast carcinoma (Supernat et al., 2012), gastric carcinoma (Lu et al., 2013), ovarian carcinoma (Oh et al., 2012) and bladder cancer (Wettstein et al., 2015). Similarly, loss of CD73 promoted tumor progression in endometrial carcinoma by eliminating adenosine-mediated protection of epithelial integrity (Bowser et al., 2016).

While most of the CD73 is a cell surface-linked glycoprotein, CD73 can also be shed from the membrane either proteolytically or by the activity of phosphatidylinositol-specific phospholipases to liberate soluble variants of the enzyme which remain catalytically active (Heuts et al., 2012). Soluble CD73 (sCD73) was shown to have regulatory functions with respect to nucleotide blood and serum level maintenance and its presence was associated with patient outcome in metastatic melanoma (Morello et al., 2017).

Recently, evidence has emerged that NK cells, which do not express CD73 in healthy tissues, may acquire some CD73 expression under specific conditions. Data reported so far is, however, inconclusive. One such condition is induction by mesenchymal stem cells (Chatterjee et al., 2014). Co-culture of NK cells with mesenchymal stem cells induced expression of CD73 on 5-10% of NK cells. Similarly, it has been suggested that tumor-infiltrating NK cells may acquire some expression of CD73 (Young et al., 2018). Though highly variable, expression was also found on a limited number of NK cells isolated from mouse melanoma (SM1WT1) tumors (Young et al., 2018). Contrasting these reports is data by Perrot et al. who reported no significant difference between CD73 expressed on circulating NK cells and those that have infiltrated tumors (Perrot et al., 2018). Collectively, this phenomenon has not been fully validated yet, is likely tumor-specific and requires further investigation.

Based on the available evidence, the roles of CD73 in cancer are both complex and significant, from the understanding of its effect on immune cell function in solid tumors to the consequent development of targeted immunotherapies.

### The Role of CD73-Expressing Tumor Exosomes

Immunosuppressive signaling via CD73 has also been reported to occur from exosomes secreted from tumors. Exosomes are extracellular vesicles released by all cells, and are involved in cell-to-cell communication (Zhang and Grizzle, 2011). When derived from tumors, they are often involved in malignant reprogramming of immune cells (Ruivo et al., 2017). Tumor-derived exosomes were shown to express CD39 and CD73 and thus participate in the production of extracellular adenosine (Clayton et al., 2011). Evidence has also shown that tumor exosomes are capable of delivering membrane-bound CD39 and CD73 proteins to regulatory T cells (T_regs_), and potentially other immune cells, lacking the expression of either enzyme, to ultimately trigger production of adenosine (Schuler et al., 2014; Ludwig et al., 2017). Similarly, CD73 expressed on exosomes derived from activated T_regs_ was shown to be involved in these cells' immunosuppressive functions via the generation of adenosine (Smyth et al., 2013). Dendritic cells are another cell type whose expression of CD73 is upregulated following co-culture with tumor-derived exosomes, specifically in this case from prostate cancer cells (Salimu et al., 2017). With regard to NK cells, exosomes extracted from the plasma of relapsed/refractory acute myeloid leukemia patients were shown to be enriched in CD39 and CD73, and when the exosomes were incubated with the human NK cells, the lytic functions and survival of NK cells were inhibited (Hong et al., 2017). Collectively, tumor-derived exosomes are capable of inducing immunosuppressive signaling via CD73-mediated adenosine production, a mechanism that is appearing as a potentially significant therapeutic target.

## CD73 Induction Under Hypoxia

Multiple studies have shown that CD73 expression is rapidly enhanced under hypoxic conditions (Li et al., 2006; Eltzschig et al., 2009; Semenza, 2012b). The transcription factor HIF-1 was shown to have a direct mechanistic link to this activation. HIF-1 is a heterodimer consisting of HIF-1α and HIF-1β subunits. Under normoxic conditions, HIF-1α is degraded by cellular proteasomes. In hypoxic environments, however, the protein is stable and can dimerize with the HIF-1β subunit, bind to regulatory regions of target genes and trigger their expression. Functionally, HIF-1 drives a shift to increase anaerobic metabolism for cellular energy production and decreases mitochondrial oxygen consumption, supporting adaptation of cellular metabolism to conditions of oxygen deprivation. The presence of HIF-1α and HIF-1β was shown to be proportionally associated with the grade of tumor progression, a characteristic that was described for gliomas (Zagzag et al., 2000). As a result, targeting HIF-1 has been a strategy under investigation for its potential as anti-cancer therapy (Semenza, 2003).

HIF-1-induced expression of CD73 is triggered by the binding of HIF-1 to the *NT5E* gene on hypoxic cells, such as cancer cells in solid tumors. This is facilitated by the CD73 gene promoter, which contains a HIF-1-binding DNA consensus motif, 5′-CCGTG-3′ (Synnestvedt et al., 2002), and is further potentiated by the fact that oxygen diffusion is limited to 100–180 μm from the capillary to the cells (Mizokami et al., 2006). Overexpression of HIF-1α was found to be associated with tumor size and depth of invasion (Lu et al., 2013), while expression of CD73 is markedly increased in metastatic cancers.

Hypoxia was also shown to enhance the expression of the adenosine A_2B_ receptor (A_2B_R) (Lan et al., 2018), which is most highly expressed on macrophages and dendritic cells (Cekic and Linden, 2016), though recent studies have reported its overexpression in certain cancers (Mousavi et al., 2015). A_2B_R has been implicated in cancer development through agonist and antagonist treatment. It was, for example, shown that A_2B_R inhibition stunted progression of bladder cancer (Zhou et al., 2017) and the growth of colon carcinoma cells (Ma et al., 2010), while its agonism could stunt proliferation of breast cancer stem cells (Jafari et al., 2018), sensitize glioblastoma stem cells to chemotherapy treatment (Daniele et al., 2014) and inhibit growth of ovarian cancer cells (Hajiahmadi et al., 2015). HIF-1α expression was recently correlated to the overexpression of A_2B_R in human oral cancer (Kasama et al., 2015) and breast cancer (Lan et al., 2018).

HIF-1α was also shown to be implicated in adenosine signaling and in increasing the formation of intracellular adenosine. It does so by inhibiting the activity of adenosine kinase, which would otherwise re-phosphorylate adenosine to AMP intracellularly (Decking Ulrich et al., 1997). Impaired re-phosphorylation results in accumulation of elevated concentrations of intracellular adenosine, which is then transported outside of the cell where it signals on immune cells including NK cells. Hypoxia has also been reported to have roles in increasing the formation of intracellular adenosine by decreasing intracellular levels of adenosine triphosphate and increasing intracellular AMP (Kobayashi et al., 2000; Synnestvedt et al., 2002).

## Metabolic Dysfunction of Natural Killer Cells

### Metabolic Reprogramming of NK Cells Under Hypoxia

NK cells are sensitive to hypoxia. In conditions of low oxygen, NK cells show impaired cytotoxic ability which is correlated to lower expression of activating receptors NKp46, NKp30, NKp44, and NKG2D, independent of the presence of cytokines IL-2, IL-15, IL-12, or IL-21 (Balsamo et al., 2013). Although there is evidence that pre-activated NK cells are able to maintain some cytotoxic function when exposed to hypoxia (Kim et al., 2018; Moon et al., 2018), hypoxic signaling was shown to induce inhibition of a number of functional mechanisms that support NK cell anti-tumor immunity (Table 1). The various levels of oxygen concentration and physical conditions can also cause differences in activation responses seen by NK cells, with more modest responses normally seen in mild hypoxic conditions (Loeffler et al., 1991; Fink et al., 2003; Lim et al., 2015). Therefore, the specific level of oxygen in the *in vivo* environment should be considered when evaluating NK cell activation.

**Table 1:** Effects of hypoxia on NK cell function and metabolism.

**Immunomodulatory factor**	**Effect**	**References**
**Oxygen level**		
Hypoxia	Downregulation of activating NK receptors NKp46, NKp30, NKp44, and NKG2D	Balsamo et al., 2013
Hypoxia (short and long term) in combination with IL-15 + IL-18 or IL-2 priming	Inhibition of oxidative metabolism, induction of CXCL8, VEGF, and MIF, release of IFNγ, TNFα, GM-CSF, and little CCL3 and CCL5	Parodi et al., 2018
Hypoxia (short term) in combination with IL-15 priming	No change in glycolytic flux or glucose consumption, enhanced release of CCL3, CCL4, CCL5, reduction in K562 cell lysis, enhanced migration through ECM (hypoxia only), progression to late apoptosis (hypoxia only)	Velásquez et al., 2016
Chronic hypoxia (4 weeks at 1% O_2_)	1,000 × reduced proliferative capacity, reduced ATP and VEGF production, loss of CD56 driven by miR-210	Ang et al., 2010
HIF-1α	HIF-1α-deficient NK cells show impaired tumor cell killing, but promote tumor inhibition due to lower tumor infiltration and lack of presentation of VEGFR-1	Krzywinska et al., 2017
Anoxia	Reduction in NK cytotoxicity against MHC-negative mouse tumor cell line YAC-1	Loeffler et al., 1991
Hypoxia (1% O_2_)	Reduction in NK cytotoxicity against K562 cells	Fink et al., 2003
Hypoxia	Reduction in expression of NKG2D, perforin and granzyme B, decrease in cytotoxicity against multiple myeloma cells, reduction in CD16 expression; IL-2 rescued these effects	Sarkar et al., 2013
Hypoxia (0.6%)	Reduction in NK cytotoxicity against K562 only when combined with lactate; limited but not impaired degranulation against myeloma cells and no change in ADCC due to hypoxia alone	Mahaweni et al., 2018a
Hypoxia (0.6%)	NKG2A^+^KIR^−^ NK cells degranulate more than NKG2A^+^KIR^+^ cells; no difference in degranulation for KIR^−^ NK cells with or without NKG2A	Mahaweni et al., 2018b

Typically, NK cells remove abnormal cancer cells though the detection of major histocompatibility complex (MHC) Class I-deficient targets expressed on various types of cancers. Interaction of the cancer cell surface stress proteins, MHC class I chain-related molecule A and B (MICA/B) or UL16 binding protein 1-6 (ULBP-1/6) with the NKG2D receptor on NK cells also triggers NK cell cytolytic action. However, in the response to hypoxia, advanced cancers can escape NK-mediated killing through shedding of cell surface MICA and MICB molecules. This shedding has been shown to occur through the stimulation of several transmembrane metalloproteinases, including members from the a disintegrin and metalloproteinase (ADAM) enzyme family and matrix metalloproteinase (MMP) family (Menier et al., 2002; Salih et al., 2002; Barsoum et al., 2011; Baginska et al., 2013). Impairment of nitric oxide (NO) signaling is also associated with MICA/B shedding, and the accumulation of hypoxia-inducible factor-1α (HIF-1α) contributes to the immune escape by increasing ADAM10 expression to decrease surface MIC molecules on the tumor cell. Activation of NO signaling can interfere with this mechanism to prevent hypoxia-induced inhibition of NK cells (Barsoum et al., 2011). MICA/B is composed of three main extracellular domains. The main site of the proteolytic shedding of MICA/B on the surface of cancer cells is the α3 domain, while the other two α1 and α2 domains bind to the NKG2D receptor. Blocking the α3 domain using domain-specific antibodies has shown to stabilize the MICA/B on tumor cells to strongly prevent shedding and stimulate tumor immunity (Andrade et al., 2018).

In addition to MIC shedding, CD16 shedding from the NK cells by cleavage from a metalloprotease, ADAM17, also occurs after NK cell activation. Although hypoxia has been shown to only minimally alter CD16 expression (Balsamo et al., 2013), the repeated activation of CD16 causes decreases in perforin secretion and insensitivity for further activation. However, the NK cells are still capable of activation by NKG2D (Srpan et al., 2018).

Normally NK cells rely on autophagy, especially during development where autophagy is important to prevent mitochondrial damage and reactive oxygen species accumulation during NK cell maturation (López-Soto et al., 2017; Germic et al., 2019). However, in the TME, hypoxia-induced autophagy in tumor cells has been shown to impair the NK cell response and degrade NK-derived granzyme B that enters the tumor cell through endocytosis, allowing the tumor to escape NK-mediated killing. To rescue NK-mediated killing and granzyme B levels, in a recent study Beclin1 (*BECN1*) was knocked down in both melanoma and breast cancer cells and shown to inhibit tumor growth. This inhibition of Beclin1 expression increased infiltration of NK cells into the tumor (Baginska et al., 2013). Through the targeting of Beclin1, it was discovered that high levels of CCL5 also increased infiltration of NK cells into the tumor. Clinically, increased CCL5 positively correlated with NKp46 to predict an improved survival rate in melanoma patients (Mgrditchian et al., 2017; Noman et al., 2018). Therefore, by targeting autophagy through Becn1 and CCL5, NK cell filtration to the tumor could increase and prevent tumor immune escape.

One of the most common methods to rescue the NK cells from the TME's hypoxic effects is to stimulate the NK cells through the use of cytokines. Besides decreases in ligand bound NKG2D activation, hypoxia (1%) can also cause downregulation of most activating receptors, including NKG2D, NKp46, NKp30, and NKp44, and IL-2 was shown to not be able to rescue this downregulation (Balsamo et al., 2013). However, in higher levels of hypoxia/anoxia (0%), the NK cell activating receptors were not affected, but CD16, perforin, granzyme B, and NKG2D were decreased. In these higher levels, IL-2 increased NKG2D expression and cytotoxicity against multiple myeloma, but most of the NK cell surface receptor activity did not change (Sarkar et al., 2013). Under short term hypoxia (1%), NK cells that were primed with IL-15 (6 h) interestingly had an up-regulation of HIF-1α and HIF-1α glycolytic gene expression. However, changes in glycolytic flux and glucose consumption did not occur. Also, unexpectedly, hypoxia without IL-15 was able to promote more migration through Matrigel. IL-15 priming alone increased specific K562 cell lysis, but late apoptosis of K562 cells *in vitro* was not increased with priming in hypoxia compared to hypoxia alone. Therefore, short-term hypoxia promotes NK cell cytotoxicity; however, IL-15 in short term hypoxia does not necessarily have a beneficial effect (Velásquez et al., 2016). A similar transcriptional study using IL-2 priming also shows increases in hypoxia and HIF related genes for both short (16 h) and long (96 h) hypoxia. With IL-2 priming, the downregulation of interferon-γ (IFN-γ) related genes occurs in hypoxia, while genes involved in proangiogenic and prometastatic functions are upregulated. In this study, other NK-activating stimuli (IL-12 + IL-18 and IL-15 + IL-18) were also analyzed. In contrast to Velásquez et al. hypoxia did not induce macrophage migration inhibitory factor (MIF) secretion and had little CCL3, CCL4, and CCL5 secretion; however, release of IFN-γ and tumor necrosis factor α (TNF-α) were observed. These discrepancies may be due to differences in time and duration of priming as well as priming cytokines (Parodi et al., 2018). Krzywinska et al. also demonstrated that IL-2 and IL-15 could not completely restore NK cytotoxicity when cultured with YAC-1 cells in HIF-1α-knockout mice (Krzywinska et al., 2017). Other cytokine stimulations evaluated to improve on hypoxic inhibition include IL-2 activation to restore NK cell cytotoxicity against multiple myeloma (Sarkar et al., 2013), and Il-12 therapy, which has been evaluated in a mouse model of murine melanoma to inhibit angiogenesis and increase tumor cell death through severe tumor cell hypoxia. However, in this last study, NK cells were not administered along with treatment, but IL-12 helped to promote NK cell infiltration and their tumor cell killing ability (Gee et al., 1999). Currently, the effect of cytokine stimulation in hypoxia is poorly understood, as most studies involve cytokine stimulations in normoxia, but cytokines play an important role in mitigating NK cell cytotoxicity against solid tumor targets.

In hypoxic environments, HIF-1α is the main transcription factor and key regulator that coordinates the hypoxic response, and HIF-1α is detected in immune cell populations. In NK cells, HIF-1α has been shown to be required for cytokine production and target cell killing. It has also been shown that HIF-1 is able to downregulate MICA expression in osteosarcoma cells, resulting in tumor resistance to NK-mediated lysis (Yamada et al., 2012). More recently, however, the loss of HIF-1α in knockout (KO) mice inhibited tumor growth even though tumor killing was impaired. This effect was demonstrated to be from a defect in NK cell infiltration into hypoxic zones of the tumor as well as reduced levels of soluble vascular endothelial growth factor receptor 1 (sVEGFR1)-expressing NK cells. This decrease in sVEGFR1 led to increased bioavailability of VEGF that subsequently caused non-productive angiogenesis and reduced tumor growth. Therefore, HIF-1α expression in NK cells in hypoxic environments may inhibit VEGF, which then promotes productive angiogenesis and tumor growth (Krzywinska et al., 2017). Overall, there are many mechanisms involved in the immunosuppression and metabolic reprogramming of NK cells in hypoxia. As the NK cell function can vary depending on hypoxic conditions, cytokine stimulations, and other physical factors, more work is needed to understand NK cell function in these complex hypoxic tumor environments.

### Immunometabolic Dysfunction of NK Cells Mediated by CD73

Hypoxia is a potent mediator of both CD39 and CD73 ectoenzymes present on cancer cells. HIF-1, which is upregulated during hypoxia, is linked to the increased expression of CD73. This increase in CD73 allows for the accumulation of adenosine from AMP in the TME which can then suppress the immune response. The immunosuppressive effects of CD73 on NK cells are varied (Table 2). Adenosine typically signals through four different G-protein coupled receptors, A_1_, A_2A_, A_2B_, and A_3_, and these receptors are present on many immune and cancer cells throughout the body to regulate the immune response. Extracellular adenosine signaling in the TME can act on the NK cells primarily through A_2A_R to upregulate cAMP and activate protein kinase A (PKA) to exert adverse effects on NK cell metabolism, cytokine production and killing function. The A_3_ adenosine receptor may also play a role on NK cell function and could inhibit cAMP activity, but the role of this receptor is not as well-studied (Wang and Matosevic, 2018). A more recent study has also linked the presence and function of transforming growth factor-β (TGF-β), a highly immunosuppressive cytokine, in the TME as a stimulator of CD39/CD73 expression through mTOR and HIF-1α to increase inhibition of NK cell activity (Li et al., 2017).

**Table 2:** Effects of CD73 on NK cell function and metabolism.

**Immunomodulatory factor**	**Effect**	**References**
**CD73**		
CD73-produced adenosine	Impairment in activating receptor expression and expression of metabolism-related genes; inhibition of glycolysis and NK cytotoxicity	Chambers et al., 2018b
CD73	Induced expression of CD73 on NK cells upon co-culture with mesenchymal stem cells	Chatterjee et al., 2014
CD73-produced adenosine	Inhibition of IFN-γ and TNF-α expression; impairment in cytolysis	Raskovalova et al., 2005
CD73	Anti-4-1BB therapy did not alter recruitment of NK cells in CD73^−/−^ mice	Chen et al., 2019
CD73	No change in anti-tumor effect of NK cells in CD73 KO mice	Wang et al., 2011
CD73 and A_2A_ receptor	Co-inhibition of CD73 and A_2A_ receptor enhanced intratumoral activity of NK cells	Young et al., 2016
CD73	Blocking CD73 enhanced cytotoxicity of NK cells against ovarian cancer cells *in vitro*	Häusler et al., 2014
CD73	TGF-β induced CD73 on MDSCs which in turn suppressed NK-mediated cytolysis of tumors	Li et al., 2017
CD73	Antibody-mediated blockade of CD73 enhanced anti-metastatic function of NK cells by blocking tumor growth	Stagg et al., 2012
CD73	Blockade of CD73 enhanced cytotoxicity and intra-tumoral infiltration of chimeric antigen receptor-NK cells *in vivo*	Wang et al., 2018
CD73-produced adenosine via A_2A_ receptor	Inhibition of NK cell maturation and anti-tumor activity	Young et al., 2018
CD73-produced adenosine	Inhibition of NK cell cytotoxicity	Raskovalova et al., 2006

CD73-produced adenosine can exert direct effects on the NK cells and can prevent NK cell cytotoxicity through impairment of metabolism. Elevated levels of adenosine have been shown to prevent granule exocytosis; however, this suppression is due to interaction of adenosine either intracellularly or with a different receptor than the A_1_ A_2_, A_3_, or adenosine receptors (Williams et al., 1997). Using a stable analog of adenosine, 2-chloroadenosine (CADO), the cytotoxic activity of lymphokine-activated, Ly49D crosslinking stimulated murine natural killer cells was evaluated, and the production of IFN-γ, TNF-α, granulocyte macrophage colony-stimulating factor, and macrophage inflammatory protein-1α were inhibited. These inhibitory effects were found to be from activation of protein kinase A isozyme I (PKA I) after cAMP stimulation from CADO (Lokshin et al., 2006). Human NK cells activated with IL-2 in the presence of adenosine were also assessed, and adenosine inhibited the NK cells' cytotoxic ability, cytokine production including IFN-γ and TNF-α, and the perforin-mediated and Fas ligand-mediated pathways in the NK cells. Again, adenosine receptor signaling was shown to inhibit NK cell function through PKA I (Raskovalova et al., 2005, 2006). In addition to adenosine, other purine nucleotides, including ATP, can affect immune function when stimulated with IL-2. In the presence of IL-2, both ATP and adenosine were able to maintain cytotoxic function against K562 cells; however, cell proliferation was inhibited. Both adenosine and ATP exhibited specific cellular pathway modulation as shown by inhibition of TNF, but not IFN-γ (Miller et al., 1999). NK cells also had anti-tumor responses and were reprogrammed to act on specific cellular pathways when stimulated with different cytokine priming programs, IL-12 and IL-15, or IL-15, or IL-2, in the presence of adenosine. NK cells were shown to be hyper-response when primed with IL-12 and IL-15, with much higher levels of IFN-γ expression than with IL-2 priming. Although IFN-γ was increased, lytic ability against CD73^+^ targets were not improved, supporting that granule exocytosis may be impaired by adenosine. Adenosine also caused downregulation of NKG2D with both IL-12 and IL-15 priming stimulations. IL-2 did not cause changes in NKG2D expression with adenosine; however, the NKG2D levels were already low (Chambers et al., 2018b).

Hypoxia also upregulates expression of A_2A_R, further driving NK cells toward impaired function by promoting their engagement with adenosine through A_2A_-mediated immunosuppressive signaling (Morote-Garcia et al., 2008). Deletion of A_2A_R in mice enhanced mature NK cell populations and delayed tumor control, showing A_2A_R-mediated adenosine negatively regulates anti-tumor responses (Young et al., 2018). During hypoxia, vascular adenosine production and signaling is enhanced, which leads to an increase in adenosine flux into the cell. In addition, HIF-1α inhibits the intracellular adenosine kinase to prevent re-phosphorylation of adenosine to AMP, resulting in even higher levels of intracellular adenosine (Morote-Garcia et al., 2008), which can weaken NK cell function. Using A_2A_R antagonists to improve anti-tumor effects as a combination therapy with either immune checkpoint blockade with anti-programmed cell death protein 1 (PD-1) (Mittal et al., 2014) or anti-CD73 (Young et al., 2016), significantly improved the anti-tumor response and prevented metastasis, showing that blocking adenosine signaling is a promising immunomodulatory strategy (Young et al., 2014).

## Targeting the Hypoxia-CD73 Axis in Solid Tumors to Enhance NK Cell Immunotherapy

### Hypoxia

Owing to its role in directing immune cell behavior, targeting soluble and membrane-associated forms of CD73 has emerged as a potentially significant immunotherapeutic approach to enhance treatment of solid tumors. The primary intent behind targeting CD73 is to alleviate the TME from the accumulation of immunosuppressive adenosine and, in turn, temper hypoxia-induced dysfunction of immune cells.

Upstream of the direct targeting of CD73, hypoxia has been recognized as a potent contributor to the heterogeneity in solid tumors and has, as a result, been targeted therapeutically (Barsoum et al., 2014; Chouaib et al., 2017; Ohta, 2018). Its effects on immune effectors are many: hypoxia has been recognized as an upregulator of the expression of PD-1/PD-L1 on tumor cells via HIF-1α (Noman et al., 2014), and impairing hypoxia sensitizes cancer cells to T cell checkpoint blockade with CTLA-4 and PD-1 (Jayaprakash et al., 2018). Similarly, hypoxia was shown to upregulate production of TGF-β in multiple malignancies (Furuta et al., 2015; Guo et al., 2016), thus contributing to the promotion of an immunosuppressive niche in the TME (Hasmim et al., 2013).

Pharmacologic inhibition of HIFs has been an obvious target, and a number of such inhibitors have been tested pre-clinically. However, targeting hypoxia is not straightforward, and no approved drugs exist that directly inhibit the HIF pathway (Li et al., 2018). This is largely because of the implication of HIF signaling in multiple pathways—over 800 genes have been reported to be targets of various HIFs (Schödel et al., 2011)—thereby precluding independent targeting to achieve a selective immunometabolic response.

EZN-2968 is an antisense oligonucleotide which antagonizes HIF1-α mRNA (Greenberger et al., 2008). Pilot and phase I trials of this agent have been carried out in patients with a variety of solid tumors (Jeong et al., 2014). Though remarkable responses have been observed in patients with cancers including renal cell carcinoma and hepatocellular carcinoma, more extensive clinical studies are needed, and are currently underway. Other small molecule inhibitors of HIF include YC-1 (Yeo et al., 2003, 1) and PX-478 (Koh et al., 2008), that are also currently in clinical trials. Outside of directly targeting HIFs, therapeutic targets under investigation owing to their involvement in HIF-1α expression, include topoisomerase 1, Hsp90 and thioredoxin (Wigerup et al., 2016). Interest in the use of engineered cells such as CAR-T therapies within the context of hypoxia is also being pursued (Schurich et al., 2019).

Targeting hypoxia within the context of CD73-mediated adenosine generation can also have indirect effects on NK cell function. Interestingly, oxygenation—the therapeutic effects of which have been seen as somewhat controversial—is being explored as a complement to NK-or T-cell based immunotherapies owing to evidence that it can impede immunosuppressive adenosinergic signaling events (Hatfield and Sitkovsky, 2015; Vaupel and Mayer, 2015). Supplemental oxygenation achieved by exposing fibrosarcoma-bearing mice to 60% oxygen—which mimics supplemental oxygen delivery to humans—led to the reduction of the expression of CD73, as well as CD39 on regulatory T cells (Tregs), in turn inhibiting their ability to produce adenosine (Hatfield et al., 2015). The positive anti-tumor effects of 60% hyperoxia were shown to rely on the presence of NK cells and their subsequent activation of T cell responses. Though the study showed that the positive effect of hyperoxia on the cytotoxicity of NK cells was driven by exocytosis of perforin-containing granules and by FAS-mediated cytotoxicity, the mechanistic cues underpinning the effects of hyperoxygenation on NK cells are largely unknown.

Targeting HIF-1α has also shown promising to enhance NK cell immunotherapy directly. Ni et al. (2018) found that tumor-infiltrating NK cells overexpress HIF-1α. By generating HIF-1α-deficient NK cells, they showed that impaired NK cell effector function could be restored, supported by a downregulated expression of inhibitory NK-associated checkpoint molecules. These cells utilized oxidative phosphorylation in favor of glycolysis to support their metabolic activity. Furthermore, by specifically targeting HIF-1α with small molecule inhibitors, NK cells showed the ability to mediate potent anti-tumor immunity.

### CD73

Despite a potentially favorable role of CD73 in certain cancers supported by a number of studies having shown a link between CD73 expression and positive disease course (Leone and Emens, 2018), directly inhibiting CD73 has proven to be a beneficial strategy to stunt tumor growth and progression. Directly targeting CD73 has shown promising anti-tumor effects *in vivo*, where an antibody-mediated blockade of CD73 inhibited growth of prostate tumors and the development of lung metastases (Stagg et al., 2012). However, despite successful targeting of CD73 alone, more profound responses could be obtained when CD73 targeting is used as part of combination treatments. Published studies have indicated that the most potent responses with anti-CD73 therapy are likely to come from administering CD73-blocking agents with other drugs, such as immune checkpoint inhibitors or adoptively-transferred immune cells. Studies have indeed shown that an antibody CD73 blockade can enhance immunotherapy of anti-PD-1 and anti-CTLA-4 monoclonal antibodies in mouse models of solid tumors (Allard et al., 2013). Chen et al. (2019) showed that the enhanced anti-tumor effect of anti-4-1BB immunotherapy stimulated by combination with an anti-CD73 blockade could be abrogated due to TGF-β in the TME, which sustained elevated CD73 expression on CD8^+^ T cells. Upon blocking TGF-β, the anti-tumor effects could be restored with the tumor cells showing, once again, sensitivity to anti-4-1BB and anti-CD73 therapy. When combined with anti-OX40 and mCTH-ANXA5, a prodrug targeting the protein annexin V, anti-CD73 therapy resulted in a significant decrease in tumor burden and an increase in infiltration of cytotoxic T cells (Virani et al., 2018). The role of TGF-β in upregulating the expression of CD73 on cancer cells has been well-documented (Regateiro et al., 2013), and its effects on the expression of CD73/CD39 on myeloid-derived suppressor cells were shown to occur by phosphorylation and activation of mTOR and HIF-1α, respectively (Li et al., 2017).

Four anti-CD73 antibodies are now in clinical trials. A Phase I study is currently underway evaluating the safety of CPI-006 (Corvus Pharmaceuticals), a CD73 activity-blocking antibody either alone or in combination with CPI-444, an A_2A_R inhibitor in patients with various solid tumors. A second anti-CD73 antibody, MEDI9447 (MedImmune), is being studied in a number of clinical trials both alone and in combination with other agents: A Phase 1 trial is evaluating the safety, tolerability and immunogenicity of MEDI9447 in combination with MEDI4736, an A_2A_R inhibitor, in patients with advanced solid tumors. In a Phase 1/2b study, MEDI9447 is being evaluated in combination with Durvalumab (anti-PD-L1) in patients with metastatic pancreatic cancer. It is being combined with Durvalumab, Tremelilumab (anti-CTAL-4) and MEDI0562 (anti-OX40) in a separate Phase 2 trial with patients with relapsed ovarian cancer. Elsewhere, a Phase 1/2 study is evaluating the safety and efficacy of MEDI9447 in combination with Paclitaxel, Carboplatin and Durvalumab for locally recurrent and inoperable metastatic triple-negative breast cancer. Bristol Meyers Squibb is conducting a Phase 1/2a trial testing BMS986179, a CD73-blocking monoclonal antibody, alone and in combination with Nivolumab, an anti-PD-1 monoclonal antibody marketed as Opdivo, in patients with advanced solid tumors. The fourth anti-CD73 antibody being studied clinically is SRF373 (also known as NZV930), developed by Surface Oncology. A Phase I/Ib study has been initiated evaluating the safety and efficacy of SRF373 in combination with an anti-PD-1 monoclonal antibody (PDR001) and an A_2A_R antagonist (NIR178) in patients with non-small cell lung cancer, triple negative breast cancer, pancreatic ductal adenocarcinoma, microsatellite stable colon cancer, ovarian cancer and renal cell carcinoma.

A new antibody targeting cell membrane-bound and soluble forms of CD73 was recently developed by Innate Pharma (IPH5301), who demonstrated synergistic anti-tumor effects when combined with a novel anti-CD39 antibody (IPH5201) in preclinical mouse models of melanoma and fibrosarcoma (Perrot et al., 2019). When compared to MEDI9447, IPH5301 managed to block AMP hydrolysis at the membrane of cancer cells and also the activity of soluble CD73 at least as effectively as MEDI9447, but, unlike the latter, it did not downregulate the expression of CD73 on membranes of cancer cells, implying a different mechanism of action.

Studies on the relationship between direct blockade of CD73 and NK cell function are still somewhat limited, although a clear argument that NK cells are directly involved in anti-CD73 responses is emerging. In a study by Häusler et al. (2014), combination of anti-CD39 and anti-CD73 blockade significantly enhanced the cytolytic activity of polyclonal NK cells against ovarian cancer cells SK-OV-3 and OAW-42 *in vitro*. Interestingly, the response was similar to that obtained upon blockade of A_2A_R with small molecule inhibitor SCH58261.

Recruitment of NK cells was shown to be critical to the *in vivo* anti-tumor responses of A_2A_R blockade in CD73^+^ tumors (Beavis et al., 2013). In the study, A_2A_R agonist NECA inhibited cytokine production and cytolytic activity of NK cells *in vitro*. In perforin-deficient mice, the A_2A_R blockade was inefficient in blocking metastasis of CD73^+^ tumors, indicating that NK cell deficiency was likely contributing to this response, and that control of metastasis of CD73^+^ tumors could benefit from NK cell engagement.

We have shown that inhibition of CD73 with blocking antibodies can enhance immunotherapy of solid tumors with CAR-NK cells (Wang et al., 2018). NK cells, engineered to express an NKG2D-based CAR with DAP10 and CD3ζ signaling domains promoted significant delay in tumor growth compared to treatment with CAR-NK cells or antibody alone following adoptive transfer into *in vivo* CD73^+^-tumor bearing mouse xenografts. Moreover, CD73 blockade appeared to enhance the intra-tumoral migration of CAR-NK cells, based on immunohistological data.

Antibodies remain the only approach which has been used to target CD73 in solid tumors. It is likely and perhaps expected, in light of the evidence of the nature and role of CD73 in tumor progression, that the level of expression of CD73 in various cancers could drive the strength of the NK cell response.

## Conclusions

Hypoxia is a powerful mediator of NK cell function and metabolism, as well as tumor progression. As a hallmark of TMEs, hypoxia exerts numerous roles on immunological responses and is considered a negative prognostic factor in the context of many solid tumors. NK cells are sensitive to hypoxia, and though they are able to maintain cytotoxic functions, such as limited degranulation, in certain conditions of low oxygen, their anti-tumor immunity is largely impaired. The role of hypoxia, and particularly HIFs, on NK cell function is complex and remains poorly understood. Despite that, the immunosuppressive effect of hypoxic signaling on NK cells via the HIF-dependent CD73-adenosinergic pathway represents an attractive and promising target in cancer immunotherapy. Administering agents that can either block CD73 and/or target HIFs concurrently with NK cell-based therapies is emerging as an immunotherapeutic strategy with significant potential within the setting of solid tumors. Further research is needed, however, to validate pre-clinical studies that have demonstrated effectiveness of targeting hypoxia and CD73 to enhance NK cell-based immunotherapies.

## Author Contributions

AC and SM performed literature review and wrote the manuscript.

### Conflict of Interest Statement

The authors declare that the research was conducted in the absence of any commercial or financial relationships that could be construed as a potential conflict of interest.
